# Case report: Multiple brain tuberculomas after *in vitro* fertilization, embryo transfer, and abortion

**DOI:** 10.3389/fneur.2022.971373

**Published:** 2022-09-13

**Authors:** Zhaobo Shi, Yong Sun

**Affiliations:** Neurology Department, Kaingfeng Central Hospital, Kaifeng, China

**Keywords:** multiple brain tuberculoma, *in vitro* fertilization, embryo transfer, anti-tuberculosis therapy, intrathecal injection, tuberculous meningitis

## Abstract

**Background:**

Multiple brain tuberculomas (MBT), characterized by disseminated tuberculous granulomas in the brain, is a rare disease like tuberculosis encountered after *in vitro* fertilization, embryo transfer (IVF-ET), and abortion. This study aimed to investigate the clinical characteristics, diagnostic methods, and therapeutic strategies of MBT after IVF-ET and abortion.

**Methods:**

A retrospective analysis was performed on the data of two patients who suffered from MBT after IVF-ET and abortion.

**Results:**

Both patients manifested headache and vomiting, which are the common symptoms of intracranial hypertension, accompanied by tuberculous meningitis. Besides, case 1 was affected by fever and epilepsy. In terms of imaging characteristics, T2-weighted imaging (T2WI) displayed multiple intracranial punctate or patchy high-intensity signals, some of which were presented as “target sign” or enhanced-like disseminated nodules, similar to miliary tuberculosis. Regular anti-tuberculosis therapy with isoniazid, rifampicin, pyrazinamide, and ethambutol was administered but failed to achieve a significant effect in the initial stage. The symptoms were gradually relieved, and the brain lesions in MRI were significantly alleviated after combining with intrathecal injections of isoniazid, dexamethasone, and chymotrypsin.

**Conclusions:**

*In vitro* fertilization, embryo transfer (IVF-ET) may be a risk factor for MBT, the common manifestations of which are intracranial hypertension. In addition to multiple nodular enhancement on brain MRI, the “target sign” on T2WI is likely to be another typical feature of MBT. Provided that there is no obvious effect of regular anti-tuberculosis therapy (ATT), intrathecal injections of isoniazid, dexamethasone, and chymotrypsin are considered to produce a favorable prognosis, but further studies are still needed to confirm the efficacy.

## Introduction

With the popularization of technology, *in vitro* fertilization combined with embryo transfer (IVF-ET) has become widely used in fertility treatment worldwide (1). After IVF-ET, the levels of estrogen and progesterone, which inhibit T lymphocytes and cause an increased incidence of tuberculosis (TB) infection, increase in pregnant women. In patients undergoing IVF-ET, the peak serum estrogen levels are several times higher than those in normal individuals. In addition, the administration of progesterone and glucocorticoids, a routine treatment after IVF-ET, is applied to ensure the normal growth of embryos, which is conducive to the reproduction of *Mycobacterium tuberculosis*, resulting in the susceptibility of pregnant patients to TB after IVF-ET (2).

TB remains one of the most important global infectious diseases, affecting nearly every system in the body, including the central nervous system (CNS) (3). Brain tuberculoma, a rare tuberculous granuloma of CNS presenting as solitary or multiple, can affect any part of the brain. Hitherto, few cases of multiple brain tuberculosis (MBT) are available in the literature (4, 5). Herein, we reported two cases with MBT after IVF-ET and abortion and analyzed the diagnosis and treatment process to provide a reference.

## Case presentation

### Case 1

**Figure 1:**
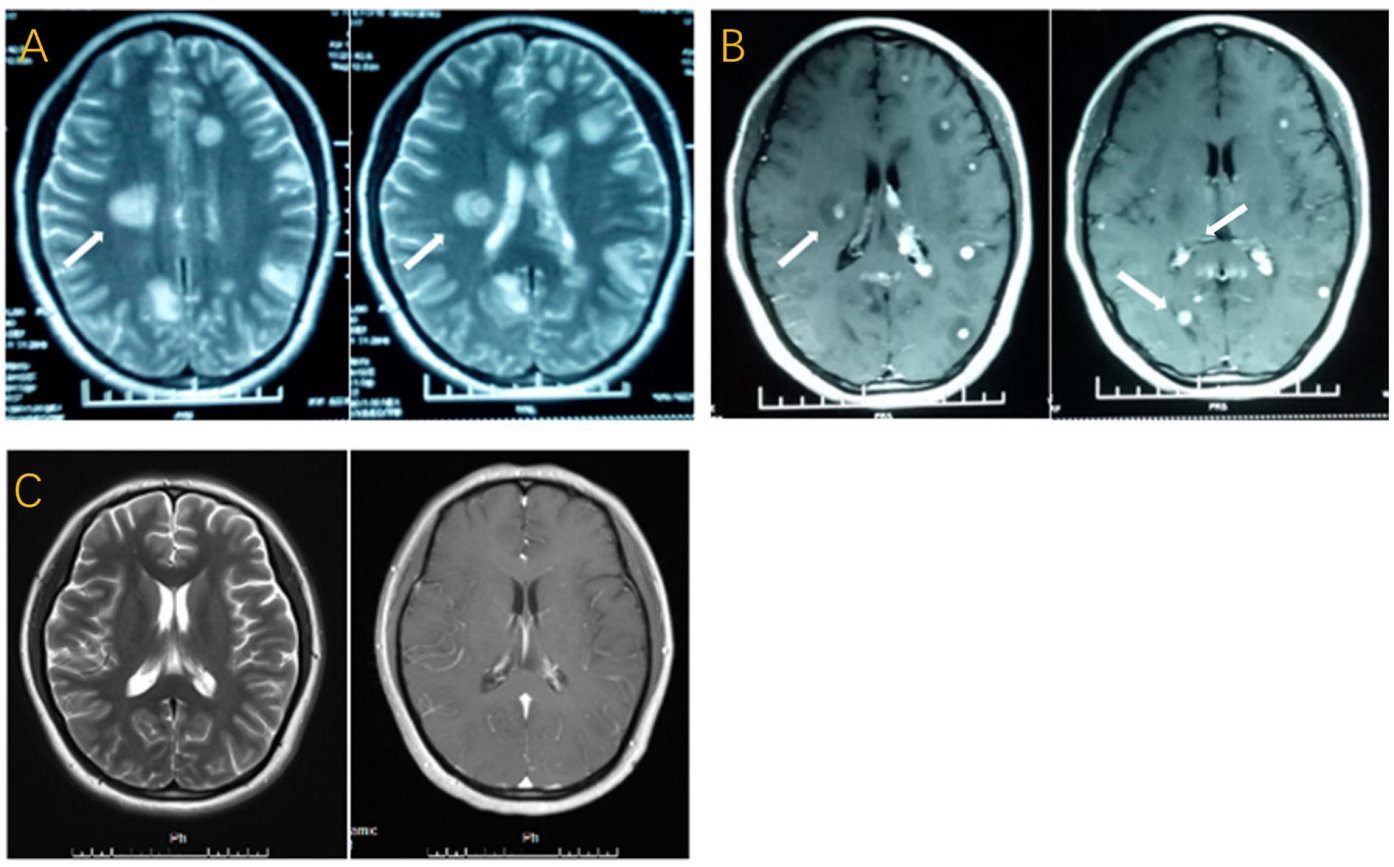
**(A)** T2-weighted image with multiple patchy high-intensity signals and “target sign”. **(B)** T1-weighted post-gadolinium contrast image with multiple nodular lesions and peripheral oedema. **(C)** T2-weighted image and T1-weighted post-gadolinium contrast image done three months later with approximate disappearance of the lesions.

A 27-year-old woman with a history of IVF-ET 7 months ago was treated with glucocorticoid, namely, 10 mg of prednisone per day for 30 consecutive days before implantation, and spontaneous abortion occurred after 4 months of pregnancy. She was previously healthy and denied any previous history of TB and close family contacts. Several days later, symptoms such as headache, vomiting, and intermittent low-grade fever developed, and she received several symptomatic treatments in the first month without any effect. Therefore, she was admitted to the neurology department at the local hospital. Figures 1A,B show multiple lesions on her brain's magnetic resonance imaging (MRI). Chest radiography was normal, and the results of a human immunodeficiency virus (HIV) test was negative. In the absence of definite etiological evidence, empirical anti-TB drugs, including isoniazid, rifampin, ethambutol, and pyrazinamide, as well as some symptomatic treatments, were given. The patient was later transferred to the First Affiliated Hospital of Zhengzhou University after her condition worsened with paroxysmal coma and seizures. The signs of meningeal irritation were positive. Lumbar puncture showed the intracranial pressure exceeding 400 mmH_2_O. CSF examination revealed low glucose (30–40 mg/dl), low chloride (119 mmol/L), WBC of 290^*^10∧9/L (65% lymphocytes and 27% neutrophils), and extremely high level of protein (297.5 mg/dl). Additionally, purified protein derivative (PPD)-positive cells accounted for 24% (reference value: <13.5%) and early secretory antigenic target (ESAT)-6 positive cells represented 23% (reference value: <9.5%) in the CSF. No mycobacteria were found in the CSF by stained smear and culture. Electroencephalogram (EEG) displayed paroxysmal spikes or sharp slow waves. No loss of consciousness with seizures occurred under the continuous administration of the previous anti-tuberculous therapy (ATT) combined with levofloxacin, prednisone, and partial symptomatic treatment, but her headache and intermittent fever were not relieved. Worse still, the patient developed herniation at the end of the first week of treatment, and she received continuous lumbar cerebrospinal fluid (CSF) drainage for one week. Based on routine treatment, intrathecal injections of isoniazid, dexamethasone, and chymotrypsin were performed every two days after removing the drainage tube. After another two months of treatment, the symptoms of fever, headache, and epilepsy were significantly relieved. The improvement of brain MRI is shown in Figure 1C. Her intracranial pressure decreased to 190 mmH_2_0. CSF examination showed reduced WBC of 30^*^10∧9/L (79% lymphocytes), significantly decreased protein (99.5 mg/dl), and normal glucose and chloride.

### Case 2

**Figure 2:**
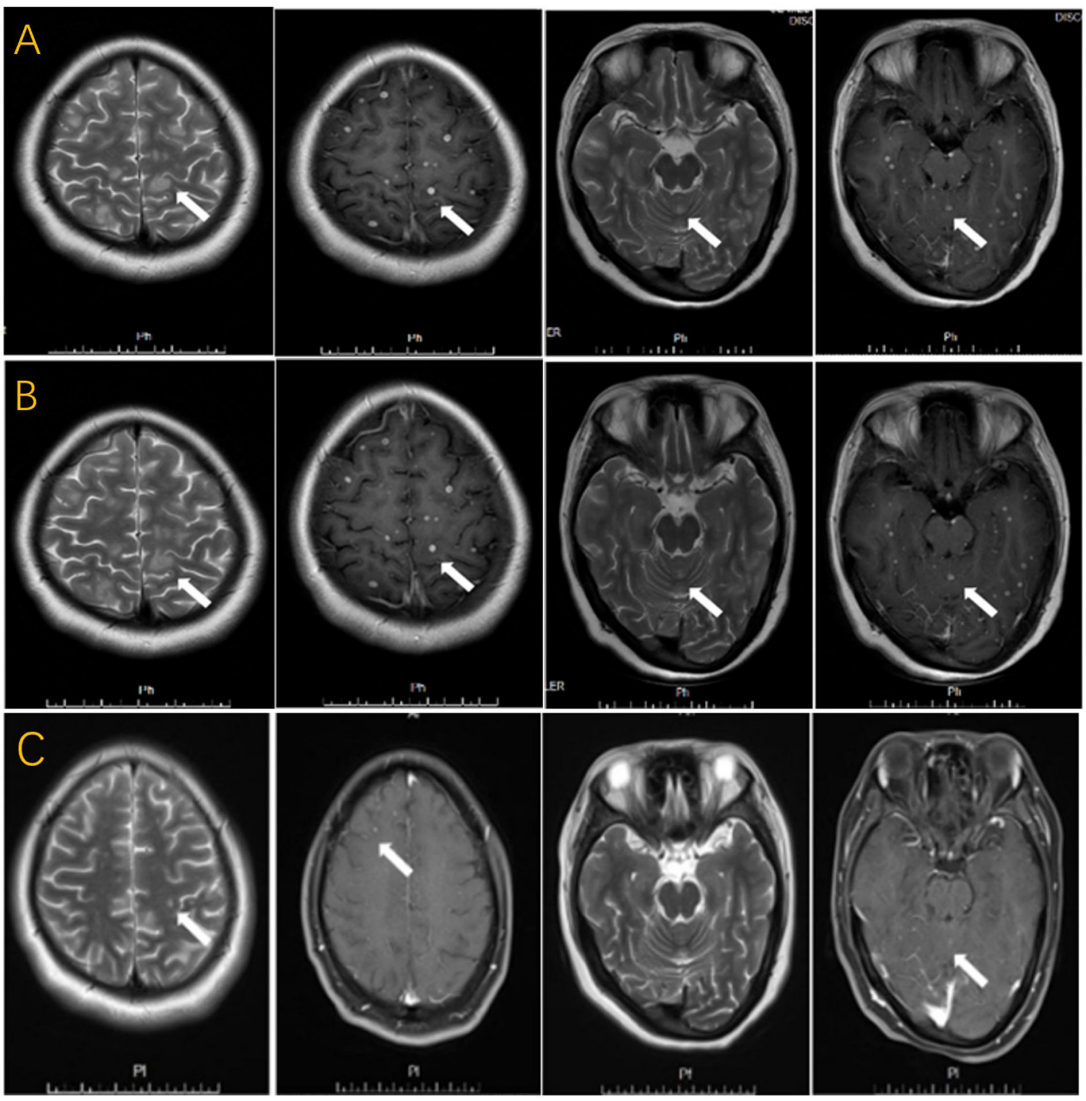
**(A)** T2-weighted image with multiple patchy hyper-intensity signals and “target sign”. T1-weighted post-gadolinium contrast image with multiple nodular enhancing lesions, similar to military tuberculosis. **(B)** T2-weighted image and T1-weighted post-gadolinium contrast image done after regular ATT for a month with no significant improvement. **(C)** T2-weighted image and T1-weighted post-gadolinium contrast image done after intrathecal injections for one month with significant decreased lesions.

A 27-year-old woman with a history of IVF-ET six months ago received 10 mg of prednisone per day for 20 days before embryo transfer. She suffered from spontaneous abortion one month ago and had a high fever immediately after the abortion. She was previously healthy and denied any previous history of TB and close family contacts. She was diagnosed with interstitial pneumonia and cured at a local hospital. Two weeks after the cure, she suddenly suffered from headaches, vomiting, and insanity. The signs of meningeal irritation were positive. In terms of auxiliary examination, CT showed no lung abnormalities, while brain MRI revealed multiple lesions (Figure 2A). The result of the HIV test was negative. The lumbar puncture showed an intracranial pressure of 110 mmH_2_O. Examination of CSF demonstrated a low level of glucose (40–50 mmol/L), pleocytosis (WBC was 22^*^10∧9/L with 73% lymphocytes), and a high level of protein (121.2 mg/dl). Moreover, PPD-positive cells accounted for 26%, and ESAT-6 positive cells represented 25% of the CSF. No mycobacteria were found in the CSF by stained smear and culture. After 1 month of anti-tuberculous drugs (isoniazid, rifampicin, ethambutol, and pyrazinamide), levofloxacin, and some symptomatic treatment, the patient still had severe headaches and vomiting. Brain MRI showed no significant changes compared with the previous scan (Figure 2B). Intrathecal injections of isoniazid, dexamethasone, and chymotrypsin were performed to specifically prevent anti-TB and reduce arachnoid adhesion. Combined with the previous therapy for another month, her clinical symptoms were relieved. Moreover, the size and number of lesions were significantly reduced on brain MRI (Figure 2C).

## Discussion

It is probably not a coincidence that both cases had a history of IVF-ET followed by spontaneous abortion. Pregnancy with miliary TB is reported to be not rare (1). However, since patients with TB may not show obvious symptoms during pregnancy and since radiographic examination is limited due to the patient's condition, the proportion of those who are not diagnosed with TB during pregnancy may be as high as 40% (6). It has been reported that the TB of the reproductive system is an important factor causing tubal infertility, and 20% of female primary infertility cases are caused by TB of the reproductive system (7). Unfortunately, whether these two patients suffered from occult TB before IVF-ET remains unclear.

Brain tuberculoma accounts for approximately 2% of CNS-TB, and brain tuberculoma with tuberculous meningitis accounts for only 10% (8). Both patients had concurrent MBT and tuberculous meningitis. Increased intracranial pressure is the most prevalent symptom among these manifestations (9, 10). In addition to high intracranial pressure, Case 1 was accompanied by fever and epilepsy, which was consistent with a larger lesion in her brain. The appearance of tuberculoma on MRI varies depending on whether the granuloma was noncaseating, caseating with a solid center, or a liquid center. Multiple hypointense on T2WI with annular or nodular enhancement on T1WI after gadolinium injection are the most common manifestations (11). However, the most characteristic lesion displayed a hyperintense core on T2WI, with a hypointense rim, and there was no obvious diffusion restriction on diffusion-weighted images, which can also be called “target sign” (4, 5). MRI of both patients displayed multiple nodules, typical target signs, and nodular enhancement. Additionally, several intraventricular tuberculomas were identified on the brain MRI in Case 1, which tended to be a factor in her later herniation.

The key to most CNS-TB diagnoses rests with the proper interpretation of the spinal CSF cellular characteristics and the chemical composition of the CSF (CSF formula) combined with the visualization of mycobacteria in the CSF by stained smear or culture. During lumbar puncture, the opening pressure is usually elevated. Typically, the CSF formula shows mononucleosis with high protein and low glucose concentration (12). However, CSF examination can be completely normal in patients with MBT without tuberculous meningitis. Detection of mycobacterial DNA by polymerase chain reaction (PCR) has a sensitivity of 33–90% and a specificity of 88–100% for the diagnosis of tuberculoma, which is a promising noninvasive approach for rapid diagnosis of tuberculoma, even in the absence of meningitis and positive stained smear and culture (13, 14). We found typical examination results of the CSF formula in two cases due to their coexistence with tuberculous meningitis. Unfortunately, no mycobacteria were found in the CSF by stained smear and culture in both cases. Moreover, they did not have access to the Next Generation Sequencing (NGS) due to their economic conditions.

Once the diagnosis of intracranial tuberculoma is suspected, routine ATT should be initiated. Failure to accept ATT immediately in Case 1 may have contributed to the greater lesion and deterioration. Additionally, both patients received oral prednisone for one month since low-dose corticosteroids are recommended to reduce brain inflammation and swelling in patients with tuberculous meningitis (15). Intrathecal injections of anti-tuberculosis drugs and anti-adhesion drugs are supposed to be beneficial for patients with MBT, especially those suffering from intraventricular tuberculoma, and MRI is a favorable approach to follow up the curative efficacy (16–18). The symptoms of the two patients did not improve significantly in the first 1 to 3 months, but the clinical conditions of the two patients improved remarkably after intrathecal injection of isoniazid against anti-tuberculosis, dexamethasone, and chymotrypsin against anti-adhesion.

## Conclusion

*In vitro* fertilization combined with embryo transfer (IVF-ET) may be a risk factor for MBT, which is often manifested as intracranial hypertension. In addition to multi-nodule enhancement on brain MRI, the “target sign” on T2WI is likely to be another typical feature of MBT. Intrathecal injection of isoniazid, dexamethasone, and chymotrypsin has a favorable prognosis on the condition of ineffective conventional anti-tuberculosis therapy (ATT). However, the efficacy needs to be confirmed in further studies.

## Data availability statement

The raw data supporting the conclusions of this article will be made available by the authors, without undue reservation.

## Ethics statement

Written informed consent was obtained from the individual(s) for the publication of any potentially identifiable images or data included in this article.

## Author contributions

ZS was the primary doctor of both cases and responsible for the writing of the manuscript and guided the completion of this article. YS contributed to the data collection and image processing. All authors contributed to the article and approved the submitted version.

## Conflict of interest

The authors declare that the research was conducted in the absence of any commercial or financial relationships that could be construed as a potential conflict of interest.

## Publisher's note

All claims expressed in this article are solely those of the authors and do not necessarily represent those of their affiliated organizations, or those of the publisher, the editors and the reviewers. Any product that may be evaluated in this article, or claim that may be made by its manufacturer, is not guaranteed or endorsed by the publisher.
